# Characterizing expanded access and compassionate use programs for experimental drugs

**DOI:** 10.1186/s13104-017-2687-5

**Published:** 2017-07-28

**Authors:** Jennifer E. Miller, Joseph S. Ross, Kenneth I. Moch, Arthur L. Caplan

**Affiliations:** 1 Division of Medical Ethics, Department of Population Health, NYU School of Medicine, 227 East 30th Street, Office 723, New York, NY 10016 USA; 2 Bioethics International, New York, USA; 3 Section of General Internal Medicine and Robert Wood Johnson Foundation Clinical Scholars Program, Department of Medicine, Yale School of Medicine, New Haven, USA; 4 Department of Health Policy and Management, Yale School of Public Health, New Haven, USA; 5 Center for Outcomes Research and Evaluation, Yale-New Haven Health, 789 Howard Ave, New Haven, CT 06519 USA; 6 Cognition Therapeutics, Inc., 2403 Sidney St # 261, Pittsburgh, PA 15203 USA; 7 Division of Medical Ethics, Department of Population Health, NYU School of Medicine, 227 East 30th Street, Office 722, New York, NY 10016 USA

**Keywords:** Compassionate use, Expanded access, Experimental drugs, Access to medicines, Ethics, Bioethics, Policy, Right to Try Laws, 21st Century Cures Act, Pharmaceutical industry, Real-world evidence

## Abstract

**Objective:**

We sought to determine the characteristics of “expanded access” and “compassionate use” programs registered in ClinicalTrials.gov and to determine the percentage of drugs provided through these programs that ultimately received FDA marketing approval.

**Results:**

We identified 398 expanded access and compassionate use programs (hereafter referred to as expanded access programs) registered on ClinicalTrials.gov. Industry funded 61% (n = 241) of programs individually or collaboratively, while NIH and the US Federal Government rarely funded programs (3% [n = 11] and 2% [n = 6], respectively). Most programs provided access to drugs (71% [n = 282]), 11% to biologics (n = 43), and 10% to medical devices (n = 40). These programs covered 460 unique conditions, the most common being HIV (n = 26), leukemia (22), and multiple myeloma (n = 14). Only 2% of programs reported results in ClinicalTrials.gov. Most programs (82%) were open to enrolling adults and seniors (n = 326). These programs provided access to 210 unique experimental drugs, of which 76% have received FDA approval.

## Introduction

Proposed federal Right to Try legislation, advocated by the White House, [1] allows physicians to prescribe experimental therapies unapproved by the U.S. Food and Drug Administration (FDA) to terminally ill patients. Proponents say these laws, which have already passed in 36 states, are vital to providing potentially lifesaving therapies to terminally ill patients who do not qualify for clinical trials. Critics contend patients can already access experimental drugs through the FDA’s Expanded Access Program, which grants 99% of requests but depends on consent by medical product manufactures [2].

Expanded access programs, sometimes also called “compassionate use” programs, provide patients with serious or immediately life threatening diseases or conditions access to investigational products outside of clinical trials. Under the current Federal Food, Drug, and Cosmetic Act (FD&C Act), patients seeking expanded access generally need their physician to determine they have no alternative available therapy and no access to a clinical trial for their disease or condition. Additionally, the patient’s treating physician must agree to apply for expanded access on their behalf and the medical product company must agree to provide the investigational drug [3]. Currently, a company can decline a request or it can approve the request, but is not obligated to provide the drug for free [4].

Much of the scholarly literature on expanded access programs focuses on the role of the FDA [5, 6] or other regulatory bodies [7] in providing patients access to experimental drugs, or the ethics [8–10] and legal aspects of these programs [11, 12]. A few papers also explore the role of social media and patient advocacy in fostering pre-approval access [13, 14]. Very few papers provide empirical data or data-driven characterizations of expanded access programs.

The main, perhaps only, empirical paper on expanded access programs was published by the FDA. It details how many expanded access requests it received over the last ten years and their rate and handling of approvals (99% of requests were approved) [15]. In general, the literature provides little to no insight on the characteristics of these programs or whether the provided experimental drugs ever receive FDA approval.

Given the contentious debate surrounding the Right to Try laws [16] and lack of empirical information about expanded access programs in the literature, we sought to determine the characteristics of “expanded access” and “compassionate use” programs registered in ClinicalTrials.gov, the trial registry maintained by the U.S. National Institutes of Health (NIH). We also sought to determine the percentage of drugs provided through these programs that ultimately received FDA marketing approval.

## Main text

### Methods

We searched ClinicalTrials.gov to identify *all* expanded access and compassionate use programs registered by July 1, 2016. After identifying all relevant trials in the ClinicalTrials.gov database using the search terms “compassionate use” and “expanded access” we reviewed trials manually for relevance and removed duplicate trials (trials listed multiple times under the same NCT number). ClinicalTrials.gov is a publicly available registry and database maintained by the National Library of Medicine (NLM) at the National Institutes of Health (NIH), created in 1997 as a results of the Food and Drug Administration Modernization Act of 1997 (FDAMA).

For each identified program, we abstracted the program’s registration date, funding source, product type (drug, biologic or device), condition treated, patient age, number patients enrolled, whether the program was for a single or multiple patients, and reporting of trial results.

For drugs only, we determined FDA approval rates by cross-referencing the drug provided in the expanded access program with Drugs@FDA. Drugs@FDA is a public database maintained by the US FDA, listing most drug products approved by the FDA since 1939. We used descriptive statistics, such as means, proportions, and medians, and all analyses were performed using Microsoft Excel v2013 (Redmond, Washington).

## Results

We found 527 trials using our search criteria on ClinicalTrials.gov. After removing 54 duplicate trials and 75 irrelevant trials, 398 programs met our final inclusion criteria as compassionate use (CU) or expanded access (EA) programs (hereafter referred to as expanded access programs). Of these 398 programs, 306 were labeled as expanded access only programs, 49 were compassionate use only, and 43 programs used both terms. The earliest recorded program started in June of 1989. It was for “Compassionate use of tetrabenazine in the treatment of abnormal movements,” sponsored by Baylor College of Medicine.

Industry funded 61% (n = 241) of the 398 programs individually or collaboratively. In contrast, the NIH and the US Federal Government rarely funded programs (3% [n = 11] and 2% [n = 6], respectively) (Table 1). 36% of programs are classified as funded by an institution “Other” than the Industry, NIH, or US Federal Government. The “Other” category generally refers to university or academic sponsors.

**Table 1:** Characteristics of registered expanded access and compassionate use programs

Characteristics	No. of programs (%)
Sponsor: individual or collaborative sponsor (N = 398 programs)	
Industry	241 (60.6)
NIH	11 (2.7)
U.S. Fed	6 (1.5)
Other^a^	143 (36)
Interventions provided (N = 398 programs)	
Drug	282 (71)
Biological	43 (11)
Device	40 (10)
Other	31 (8)
Missing data	2 (.5)
Conditions treated^b^ (N = 460)	
HIV	26 (6.5)
Leukemia	22 (5.5)
Multiple myeloma	14 (3.5)
Cholestasis	12 (3)
Melanoma	11 (2.7)
Diabetes	11 (2.7)
Lymphoma	9 (2.2)
Neuroblastoma	7 (1.7)
Age group (N = 398 programs)	
Adult only	20 (5)
Adult/senior	215 (54)
Child only	25 (6.3)
Adult|child	26 (6.5)
Child|adult|senior	111 (27.9)
Senior only	1 (.3)
Single vs. multi-patient program (N = 398 programs)	
Single-patient program	3 (.75)
Multiple patient program	73 (18.3)
Missing data	322 (80.9)
Report results (N = 398 programs)	
Results reported	8 (2%)
No results reported	390 (98)
FDA approval rates of provided drugs (N = 282 programs providing drugs)	
Drug was FDA approved	192 (68)
Drug was not approved	76 (27)
Insufficient information or uncertain	14 (5)

^a^Generally, academic institutions

^b^Top 8 conditions with the most programs

Most programs [71% (n = 282)] provided access to drugs, 11% biologics (n = 43), and 10% medical devices (n = 40). These programs covered 460 unique conditions, the most common being HIV (n = 26), leukemia (22), and multiple myeloma (n = 14). Most programs (82%) were open to enrolling adults and seniors (n = 326). Several registration fields lacked data. For example, 80% didn’t indicate whether the program was single-patient or multi-patient, 74% lacked phase data, and 98% had no reported results (n = 390).

The FDA subsequently granted marketing approval for drugs provided in 68% (n = 192) of expanded access programs. These programs provided access to 210 unique experimental drugs, of which 76% (n = 160 of 210) have received FDA approval. We did not analyze FDA approval rates for the biologics or devices.

## Discussion

Nearly 400 expanded access and compassionate use programs were registered on ClinicalTrials.gov as of July 2016. Most programs were sponsored by medical product manufacturers. This suggests that even without federal Right to Try legislation, the pharmaceutical industry is establishing programs to make experimental therapies available to terminally ill patients.

Most (76%) provided drugs in expanded access programs eventually received FDA approval. Thus, provided drugs in *registered* expanded access programs are, more times than not, eventually deemed safe by the FDA. Notwithstanding, the fact that nearly 25% of expanded access drugs have yet to receive FDA approval, shows that we cannot entirely eliminate safety and efficacy questions in expanded access and compassionate use programs.

It is reasonable to allow the FDA to retain its oversight and approval role for these programs, in order to help mitigate safety risks for patients- especially since it approves 99% of expanded access and compassionate use requests. Currently, patients wishing to gain access to experimental drugs through compassionate use and expanded access programs, must obtain FDA approval. Proposed Right to Try Legislation generally removes this step of needing FDA oversight and approval.

Legislative efforts should also aim to expand patient access to clinical trials, which in some cases could alleviate the need for expanded access and compassionate use programs. Currently, clinical trial access is often limited by several factors, including a patient’s location, age, and health status [17]. Most clinical trials enroll younger, healthier, and whiter patients than the typical patient population [18–20]. The 21st Century Cures Act makes some progress in expanding access to clinical trials.

Expanded access programs raise broader ethical and regulatory questions, including whether (and how much) product manufactures should re-direct investigational products and resources from formal clinical trials to patients requesting expanded access and how to finance these programs. In addition, experts are using [21] or advocating the use [22] of expanded access programs as “real world evidence” for drug safety or efficacy. While the reliability of using these data for this purpose is debatable, it’s worth noting that, thus far, we found only 2% of programs reported results in ClinicalTrials.gov.

## Limitations

Important information about expanded access and compassionate use programs was frequently missing from ClinicalTrials.gov, limiting precise insight into exactly which patients and how many are accessing experimental agents. This limitation should decrease over time. The FDA Amendments Act (FDAAA) final rule, released in September 2016, newly requires expanded access programs be registered on ClinicalTrials.gov and be kept up-to-date. Thus, ClinicalTrials.gov should improve in robustness over time for understanding U.S. expanded access programs.

## Abbreviations

FDA: U.S. Food and Drug Administration
FD&C Act: Federal Food, Drug, and Cosmetic Act
NIH: U.S. National Institutes of Health
NLM: National Library of Medicine
FDAMA: Food and Drug Administration Modernization Act of 1997
FDAAA: FDA Amendments Act

## References

[1] White House. Readout of the vice president’s meeting with right to try advocates. https://www.whitehouse.gov/the-press-office/2017/02/07/readout-vice-presidents-meeting-right-try-advocates. Accessed 15 Feb 2017.

[2] U.S. Food and Drug Administration. Expanded access INDs and protocols 2009–2015. http://www.fda.gov/NewsEvents/PublicHealthFocus/ExpandedAccessCompassionateUse/ucm443572.htm. Accessed 15 Feb 2017.

[3] U.S. Food and Drug Administration. Expanded access (compassionate use) https://www.fda.gov/NewsEvents/PublicHealthFocus/ExpandedAccessCompassionateUse/default.htm. Accessed 23 June 2017.

[4] U.S. Food and Drug Administration. Charging for investigational drugs under an IND—questions and answers, guidance for industry. https://www.fda.gov/downloads/Drugs/GuidanceComplianceRegulatoryInformation/Guidances/UCM351264.pdf Accessed 23 June 2017.

[5] Jarow JP, Lurie P, Ikenberry SC, Lemery S (2017). Overview of FDA’s expanded access program for investigational drugs. Ther Innov Regul Sci.

[6] Falit BP, Gross CP (2008). Access to experimental drugs for terminally ill patients. JAMA.

[7] Whitfield K, Huemer K-H, Winter D (2010). Compassionate use of interventions: results of a European Clinical Research Infrastructures Network (ECRIN) survey of ten European countries. Trials.

[8] Caplan A, Ray A (2016). The ethical challenges of compassionate use. JAMA.

[9] Welch MJ, Lally R, Miller JE (2015). The ethics and regulatory landscape of including vulnerable populations in pragmatic clinical trials. Clin Trials.

[10] Bunnik EM, Aarts N, van de Vathorst S (2017). The changing landscape of expanded access to investigational drugs for patients with unmet medical needs: ethical implications. J Pharm Policy Pract.

[11] Darrow JJ, Sarpatwari JD, Avorn J, Kesselheim AS (2015). Practical, legal, and ethical issues in expanded access to investigational drugs. N Engl J Med.

[12] Zettler PJ, Greely HT (2014). The strange allure of state “right-to-try” laws. JAMA Intern Med.

[13] Mackey TK, Schoenfeld VJ (2016). Going “social” to access experimental and potentially life-saving treatment: an assessment of the policy and online patient advocacy environment for expanded access. BMC Med.

[14] Zettler PJ (2015). Compassionate use of experimental therapies: who should decide?. EMBO Mol Med.

[15] Jarow JP, Lemery S, Bugin K, Lowy N (2017). Ten-year experience for the Center for Drug Evaluation and Research, Part 2: FDA’s role in ensuring patient safety. Ther Innov Regul Sci.

[16] Friedlaender J. The proposed federal ‘Right-To-Try’ law is not the answer for critically ill patients. Health Affairs Blog. http://healthaffairs.org/blog/2016/09/27/the-proposed-federal-right-to-try-law-is-not-the-answer-for-critically-ill-patients/. Accessed 15 Feb 2017.

[17] Downing NS, Shah ND, Neiman JH, Aminawung JA, Krumholz HM, Ross JS (2016). Participation of the elderly, women, and minorities in pivotal trials supporting 2011–2013 U.S. Food and Drug Administration approvals. Trials.

[18] Downing NS, Aminawung JA, Shah ND, Krumholz HM, Ross JS (2014). Clinical trial evidence supporting FDA approval of novel therapeutic agents, 2005–2012. JAMA.

[19] Gifford AL, Cunningham WE, Heslin KC, Andersen RM, Nakazono T, Lieu DK (2002). Participation in research and access to experimental treatments by HIV-infected patients. N Engl J Med.

[20] Heiat A, Gross CP, Krumholz HM (2002). Representation of the elderly, women, and minorities in heart failure clinical trials. Arch Intern Med.

[21] Ascierto PA, Simeone E, Sileni VC (2014). Clinical experience with ipilimumab 3 mg/kg: real-world efficacy and safety data from an expanded access programme cohort. J Transl Med.

[22] Fromer MJ. Road to successful use of real-world evidence for drug development is long and rocky, ASCO report. http://www.ascopost.com/issues/july-25-2016/road-to-successful-use-of-real-world-evidence-for-drug-development-is-long-and-rocky. Accessed 15 Feb 2017.

